# Supplementary material for: Identification and validation of novel microtubule suppressors with an imidazopyridine scaffold through structure-based virtual screening and docking
Source: RSC Med Chem. 2022 May 18;13(8):929–43. doi: 10.1039/d1md00392e (PMC9384815; doi:10.1039/d1md00392e)
Supplement: MD-013-D1MD00392E-s001 [file MD-013-D1MD00392E-s001.pdf]

Electronic Supplementary Material (ESI) for RSC Medicinal Chemistry.
This journal is © The Royal Society of Chemistry 2022

ELECTRONIC SUPPORTING INFORMATION

Identification and Validation of Novel Microtubule Suppressors with an Imidazopyridine Scaffold through Structure-Based Virtual Screening and Docking.

Samia A. Elseginy,[1,2], A. Sofia F. Oliveira,[1,3] Deborah K. Shoemark[1], Richard B. Sessions[1*]

[1]School of Biochemistry, University of Bristol, Biomedical Sciences Building, University Walk, Bristol, BS8 1TD, U.K.

[2]Green Chemistry Department, Chemical Industries Research Division, National Research Centre, 12622, Egypt.

[3]School of Chemistry, University of Bristol, Bristol.BS8 1TS, UK

Table of Contents

Table S1: Chemical structures and docking energies of shortlisted compounds.

Table S2: Physical properties of shortlisted compounds.

Table S3: effect of compound 6 on phases of the MCF7 cell cycle.

Table S4: Molecular docking results for compounds **6**, **8** and **9**.

Table S5: RMSD averages of the molecular dynamics simulations.

Figure S1: Schematic view of pharmacophore structure-based virtual screening.

Figure S2: Similarity clustering of the best 61 compounds (99 from virtual screening filtered for toxicology risk) and 50 ligands from complexes of known structure.

Figure S3: Chemical and geometric properties of the best 99 compounds from virtual screening and 50 ligands from complexes of known structure.

Figure S4: RMSD plots of the molecular dynamics simulations.

Figure S5: Ligplot$^+$ protein-ligand contact diagrams.

Figures S6-S11: Compound characterisation data, ^{1}H, ^{13}C NMR, GCMS.

Table S1: Chemical structure of shortlisted compounds and their binding energies by BUDE, AutoDock, and MOE.

Cp.	ZINC-ID	Structure	BUDE binding energy kJ/mol	AutoDock binding energy Kcal/mol	MOE Binding Energy kcal/mol
1	Zinc02843810	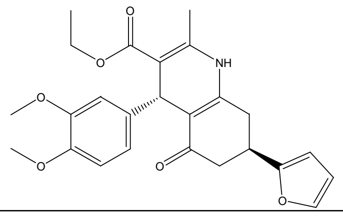	-111.013	-9.79	-6.28
2	Zinc02691641	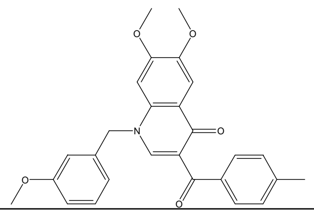	-92.82	-10.31	-7.2
3	Zinc03614688	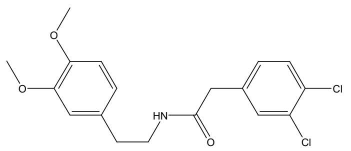	-91.78	-7.87	-5.2
4	Zinc49543397	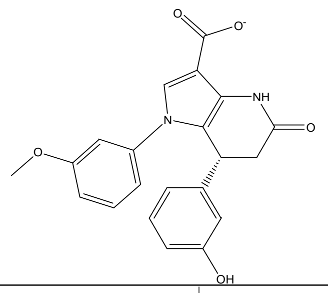	-104.3	-9.14	-8.01
5	Zinc02690781	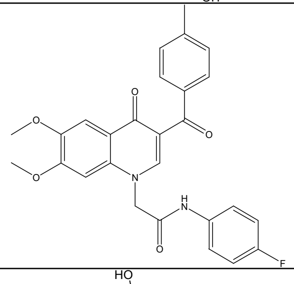	-100.75	-10.08	-8.24
6	Zinc36360243	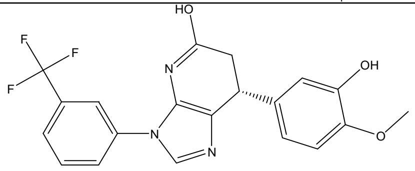	-100.9	-8.57	-10.88

7	Zinc18200970	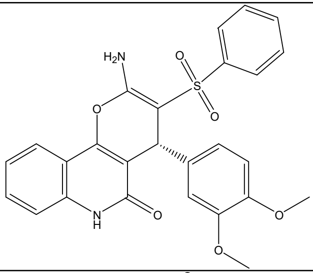	-125.35	-9.7	-6.9
8	Zinc02690805	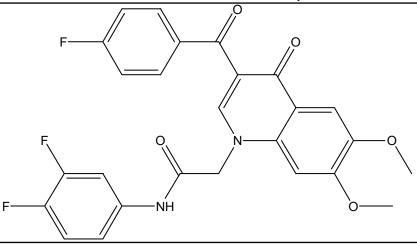	-119.48	-9.65	-9.61
9	Zinc02690789	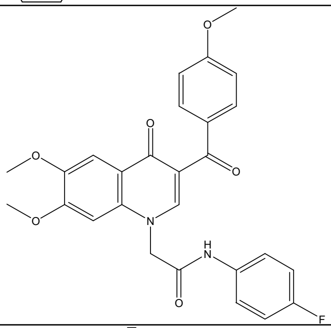	-108.21	-10.02	-8.93
10	Zinc07095120	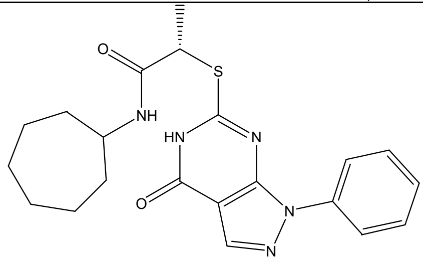	-83.65	-9.79	-5.5
11	Zinc11112053	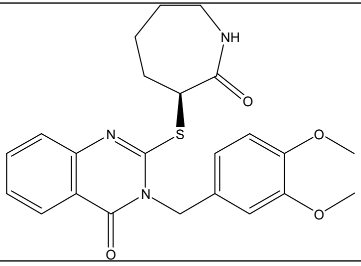	-83.66	-9.9	-6.32
12	Zinc23483881	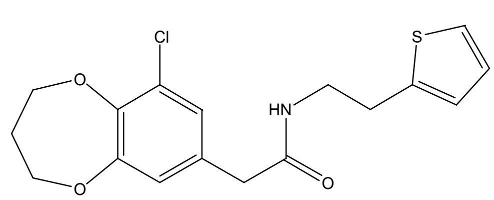	-84.55	-9.9	-5.7
13	Zinc09373064	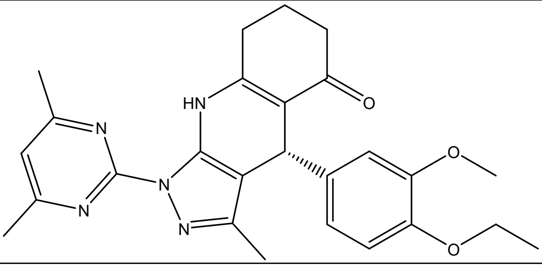	105.95	-10.91	-6.36

Table S2: Physical properties of shortlisted compounds calculated by MOE.

Compounds	ZINC ID	MwSt	Log p (O/W)	H-bond donors	H-bond acceptors	tPSA	Rotatable bond
1	Zinc02843810	437.49	2.6	1	4	87.00	7
2	Zinc02691641	443.49	4.6		5	65.07	5
3	Zinc03614688	368.26	4.08	1	3	47.56	3
4	Zinc49543397	377.37	3.16	2	3	103.62	4
5	Zinc02690781	474.48	3.79	1	5	84.94	8
6	Zinc36360243	403.36	3.9	2	5	79.87	4
7	Zinc18200970	490.53	3.4	2	5	116.95	5
8	Zinc02690805	496.44	3.8	1	5	84.94	8
9	Zinc02690789	490.48	3.4	1	6	94.17	9
10	Zinc07095120	411.53	4.05	2	4	88.38	6
11	Zinc11112053	439.53	3.7	1	5	80.23	6
12	Zinc23483881	351.85	3.3	1	3	47.56	6
13	Zinc09373064	459.53	3.2	1	6	91.16	5

Table S3 The effect of compound **6** on the phases of the MCF7 cell cycle.

compounds	%G0-G1	%S	%G2-M	%Pre G1
Control	53.91	42.71	3.38	1.48
**6**	36.28	29.66	34.06	27.36

Table S4 Hydrogen bonding of compounds **6,8** and **9** docked into the colchicine binding site.

Cp	Interacting moiety in compound	Amino acid involved	Distance Å	Type of interaction
**6**	OH-imidazopyridine<br>OH-phenyl	C=O Thrα179<br>NH Alaβ250	2.5<br>3.1	H-bond<br>H-bond
**8**	C=O<br>C=O	SH Cysβ241<br>OH Serα178	3.1<br>2.9	H-bond<br>H-bond
**9**	OCH_3<br>C=O	SH Cysβ241<br>OH Serα178	3.6<br>3.0	H-bond<br>H-bond

Table S5 Average RMSD values (Å) of complexes of **6, 8, 9, 14, 15** and colchicine with tubulin over 1.5 µs of simulation.

Å	**6**	**8**	**9**	**14**	**15**	**colchicine**
Average RMSD ligand	1.4 ± 0.3	3.4 ± 0.5	2.2 ± 0.8	2.2 ± 0.8	1.7 ± 0.3	1.5 ± 0.4
Average RMSD protein (AB)	2.5 ± 0.2	2.5 ± 0.2	2.4 ± 0.2	2.6 ± 0.3	2.6 ± 0.3	2.5 ± 0.2

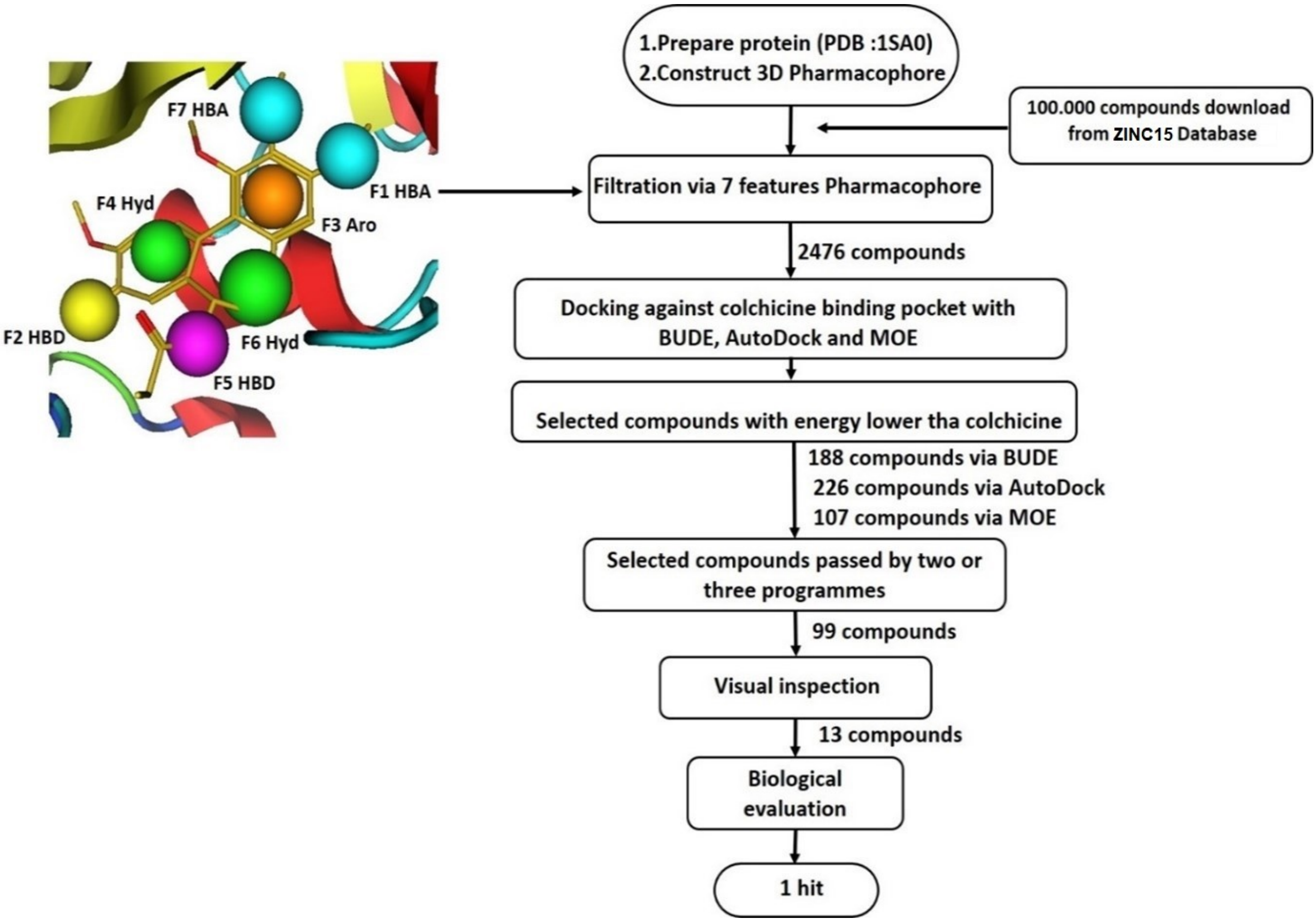

Figure S1 Schematic view of pharmacophore structure-based virtual screening.

A)

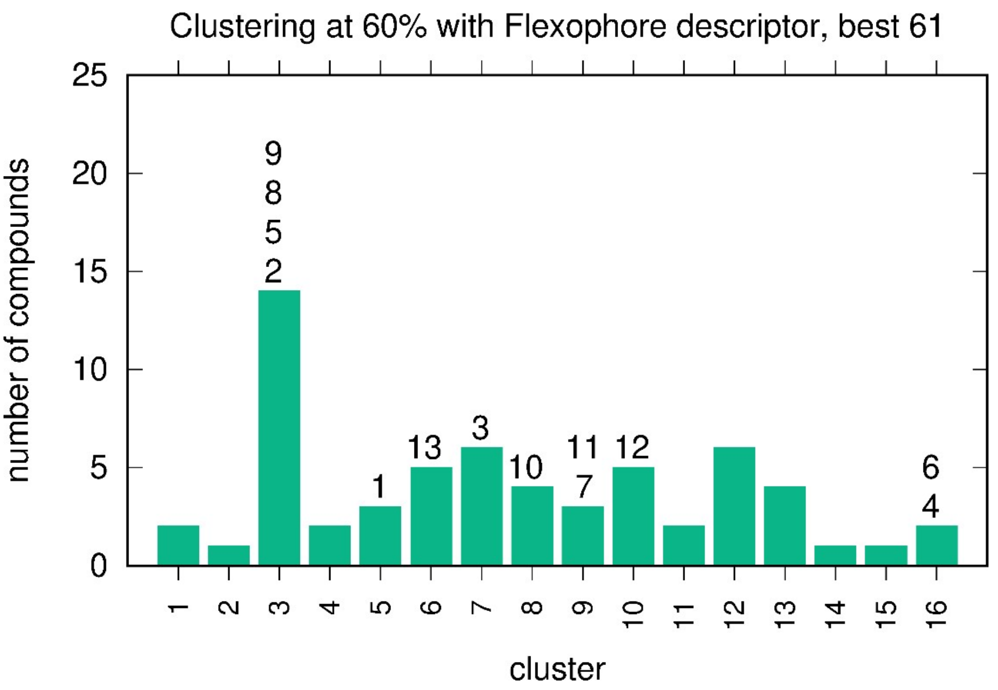

B)

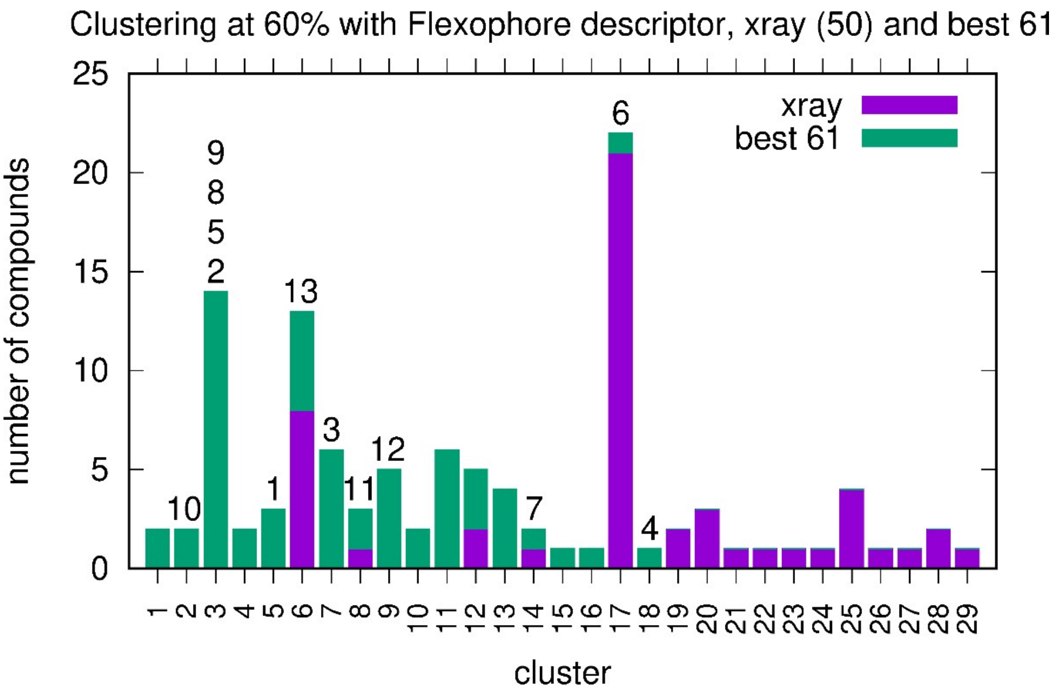

Figure S2 A) Clustering of the best 61 compounds with the Flexophore descriptor at 60% similarity. **B)** Clustering of the best 61 compounds with the 50 ligands in the colchicine site from crystal structures reported to date, using the Flexophore descriptor at 60% similarity. The cluster locations of **1-13** are indicated.

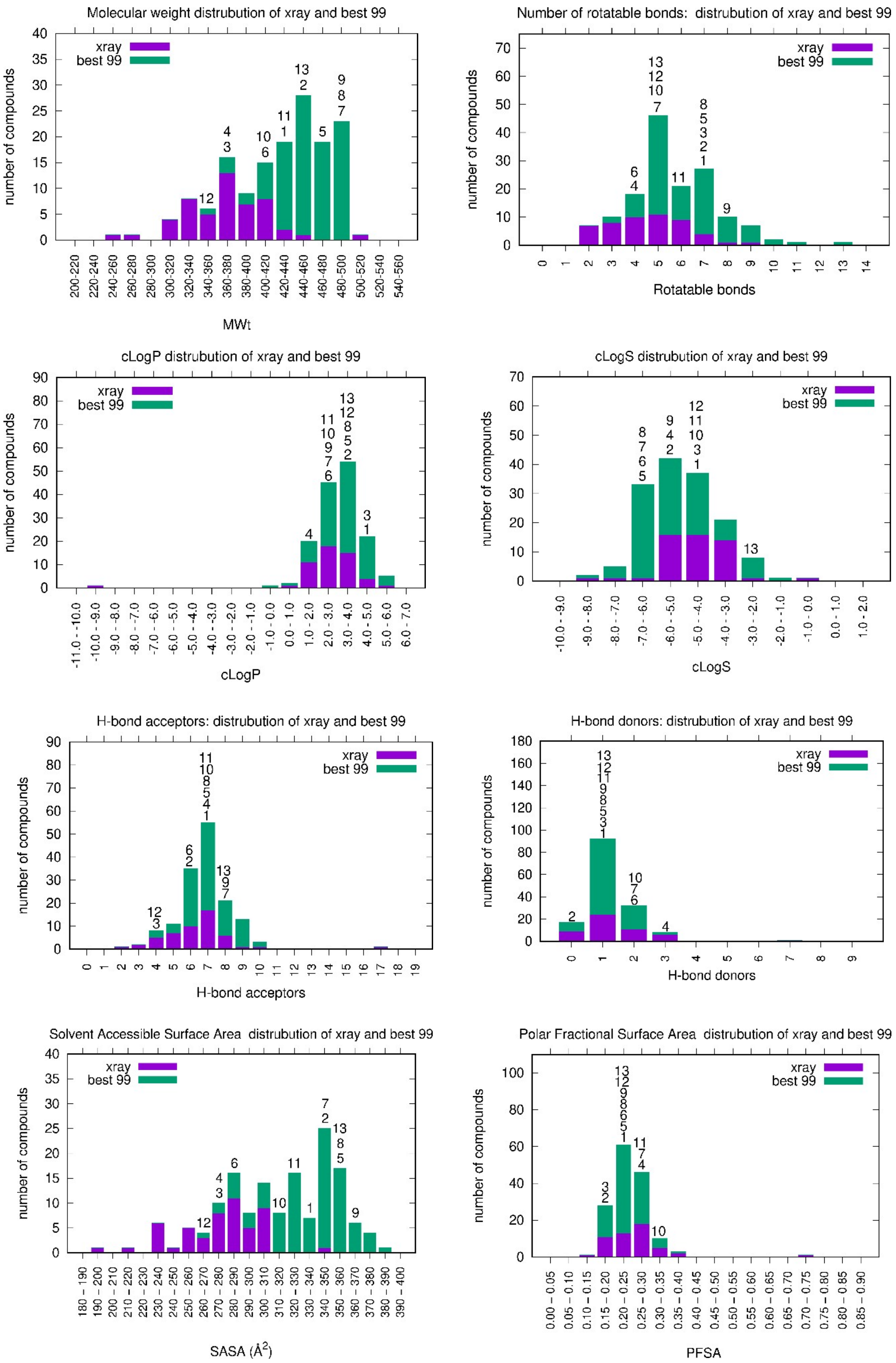

Figure S3 Histograms of ligand properties, comparing the 99 ligands from virtual screening with the 50 ligands from known crystal structures. Locations of the shortlisted compounds are indicated by the numbers.

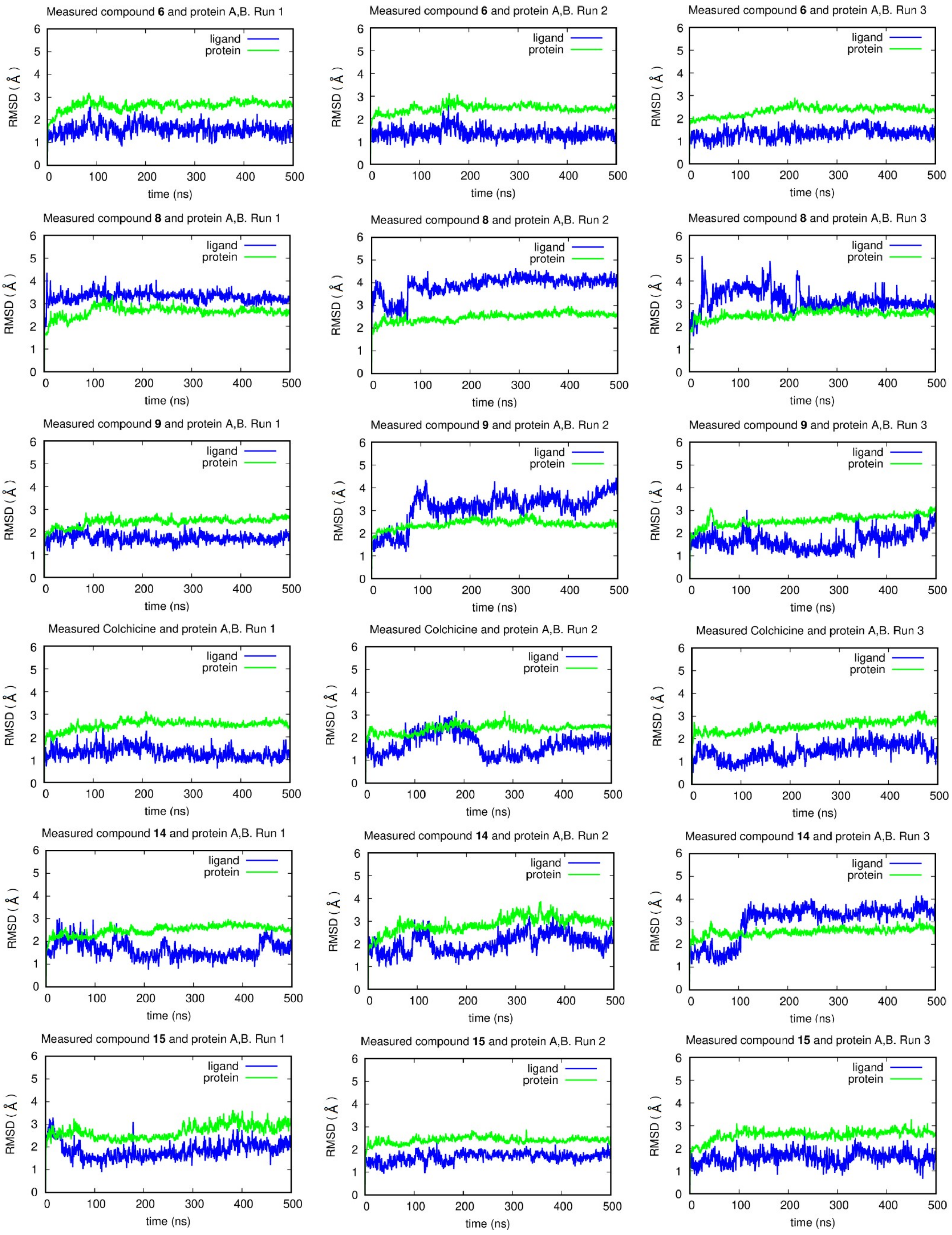

Figure S4 RMSD plots of all protein atoms of tubulin subunits A and B (α and β) superimposed on the time = 0 ns structure over the trajectories and measuring

all atoms of the tubulin protein subunits A and B (green) and all atoms of the ligand (blue).

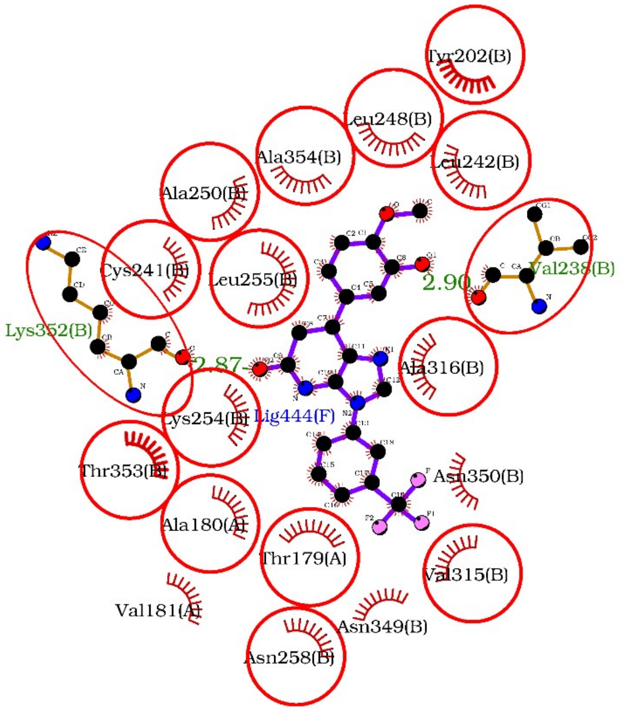

Compound 6 500 ns run 1

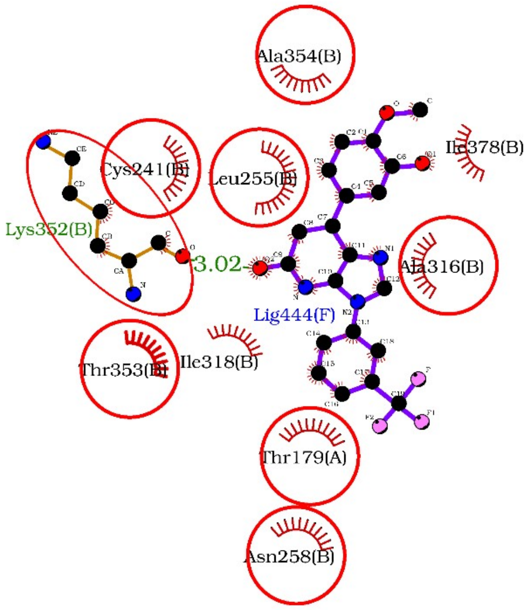

Compound 6 500 ns run 2

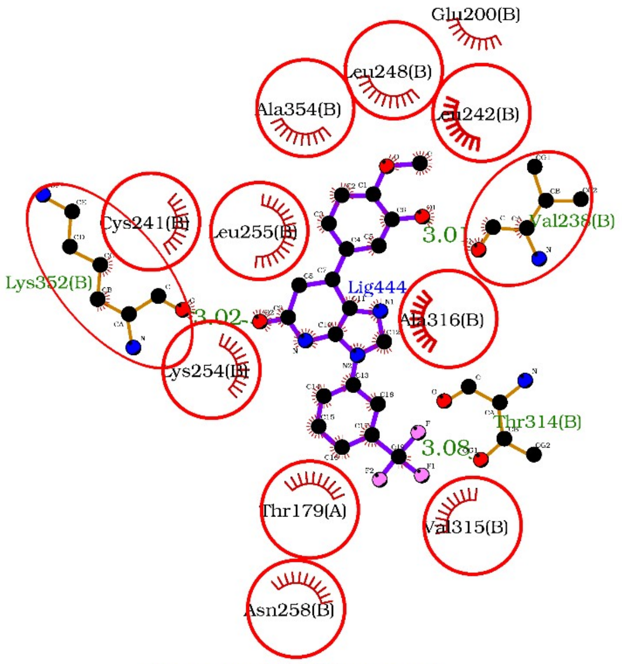

Compound 6 500 ns run 3

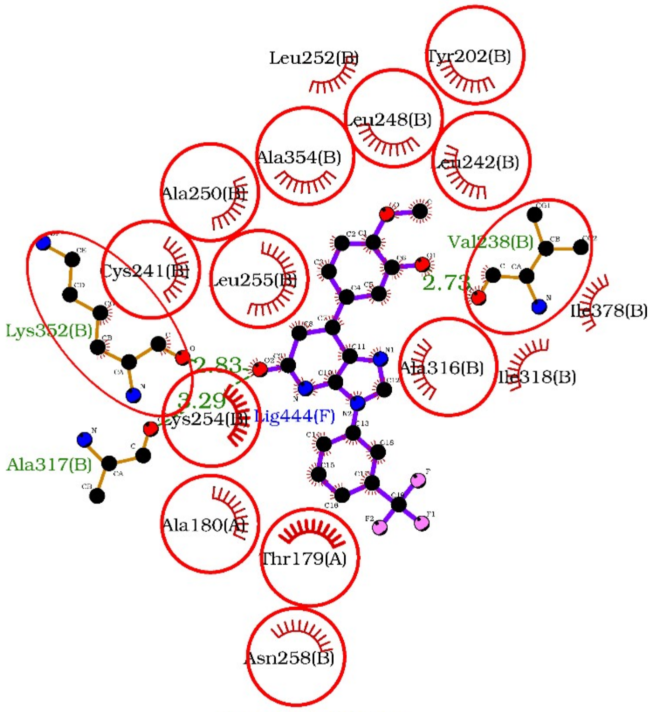

Compound 6 0 ns

.

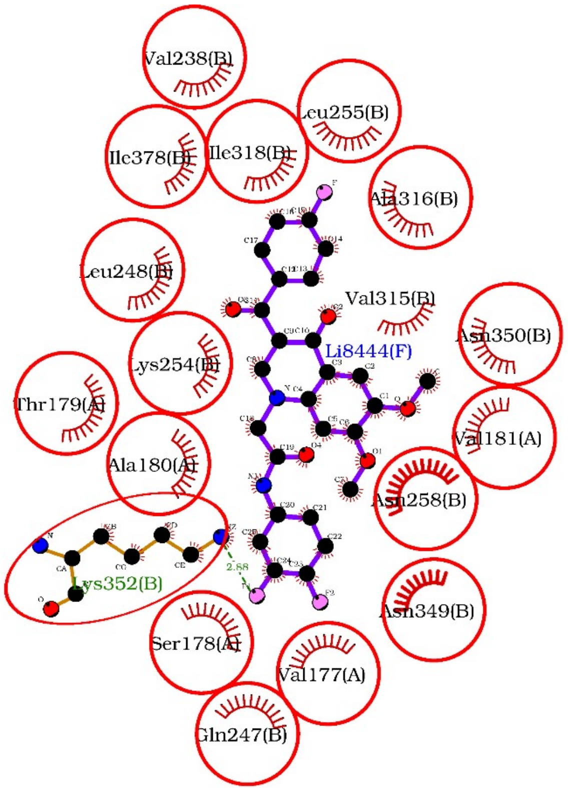

Compound 8 500 ns run 1

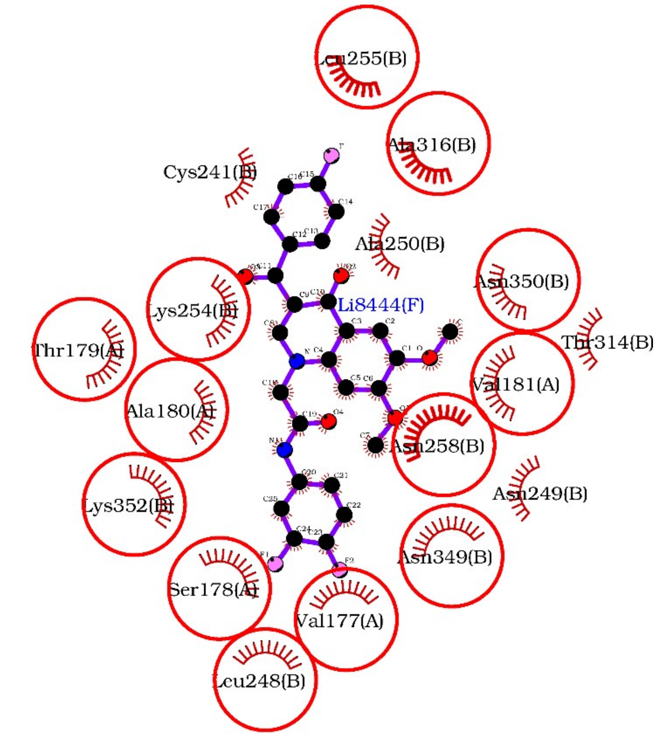

Compound 8 500 ns run 2

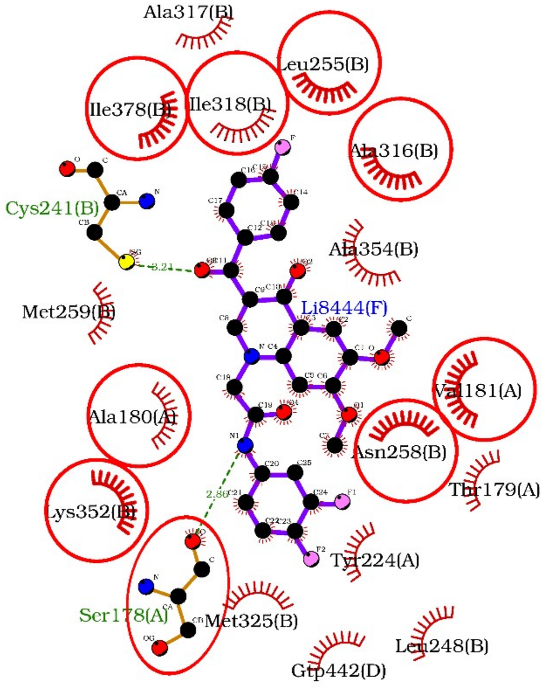

Compound 8 500 ns run 3

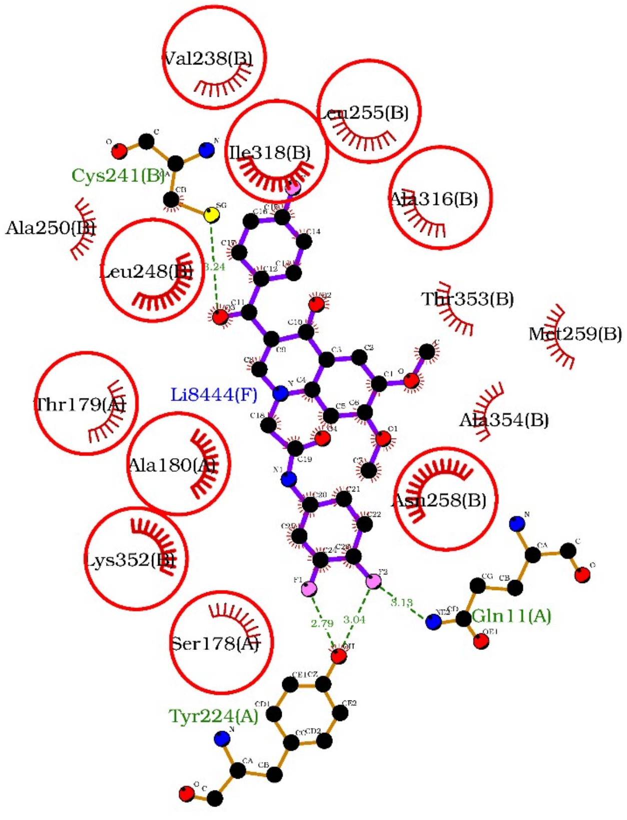

Compound 8 0 ns

.

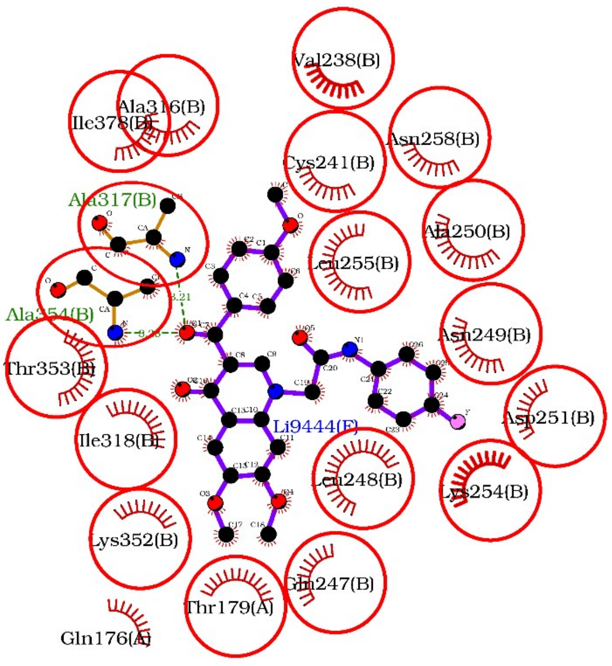

Compound 9 500 ns run 1

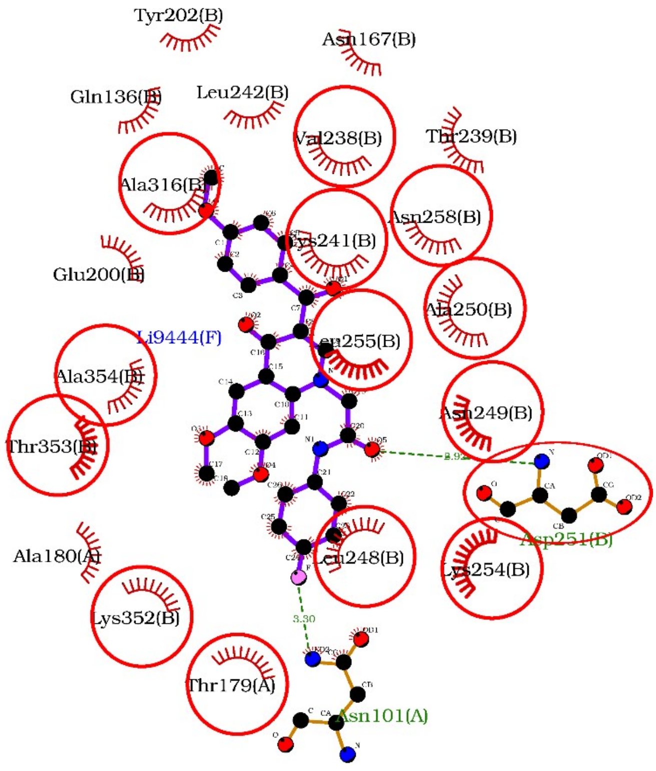

Compound 9 500 ns run 2

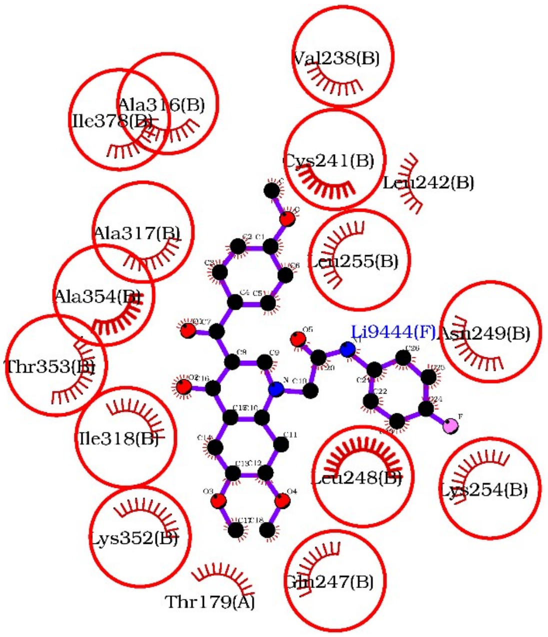

Compound 9 500 ns run 3

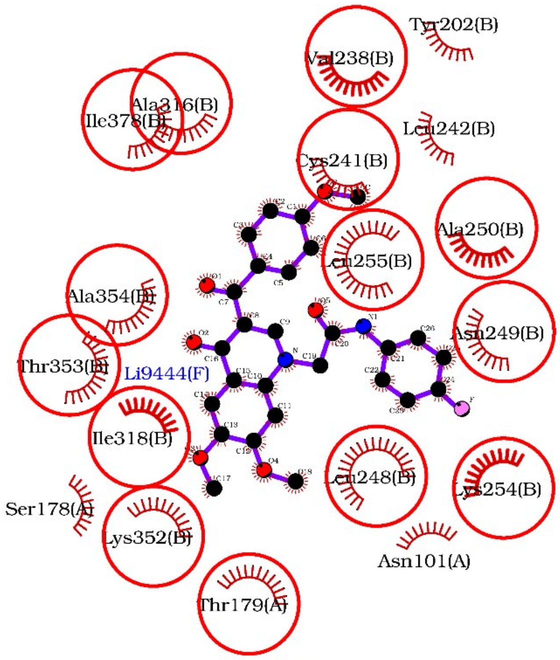

Compound 9 0 ns

.

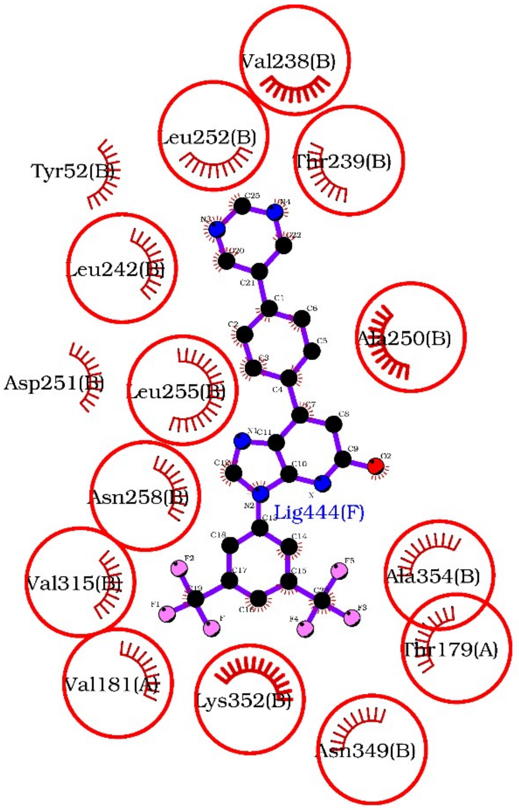

Compound 14 500 ns run 1

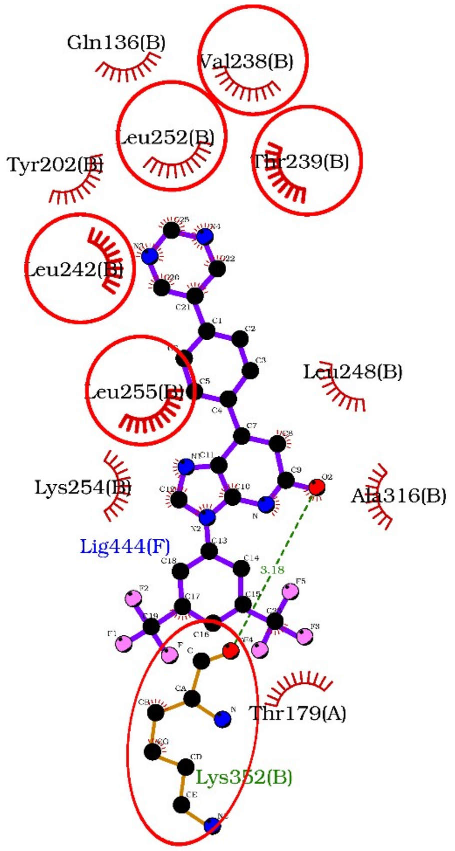

Compound 14 500 ns run 2

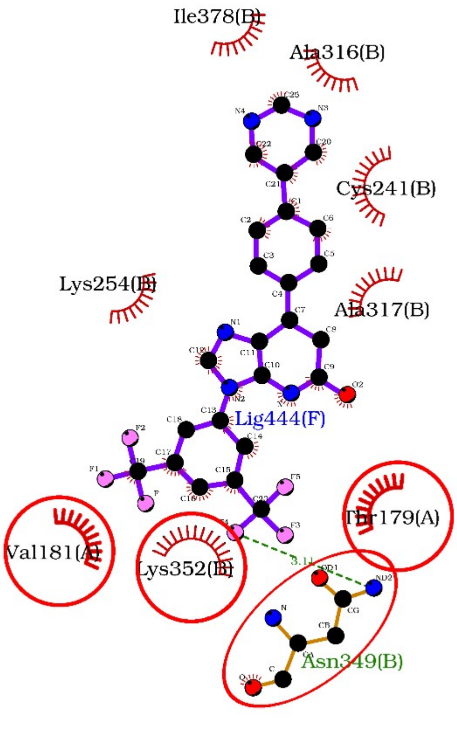

Compound 14 500 ns run 3

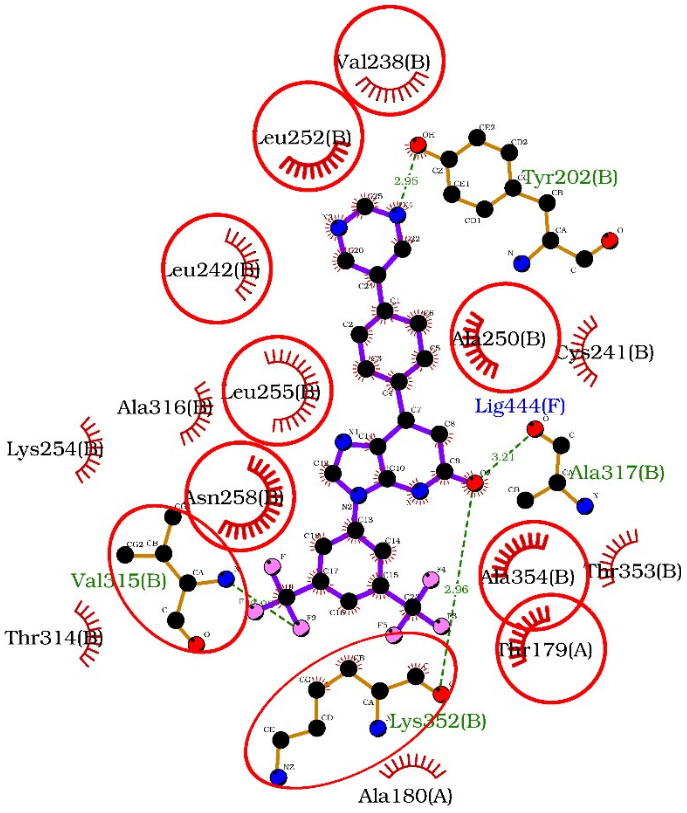

Compound 14 0 ns run 1

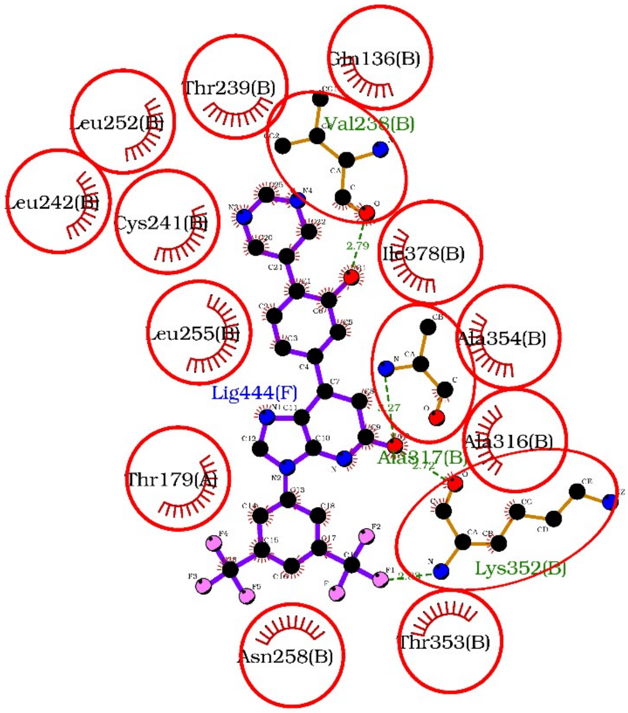

Compound 15 500 ns run 1

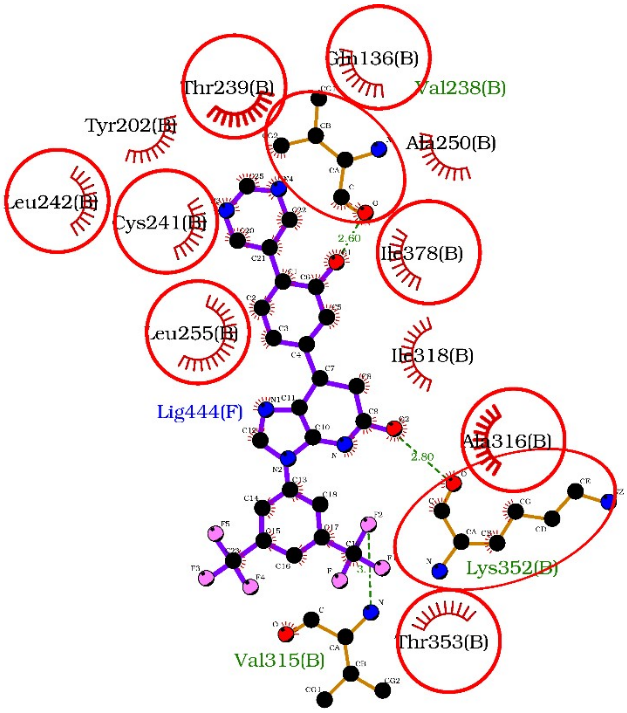

Compound 15 500 ns run 2

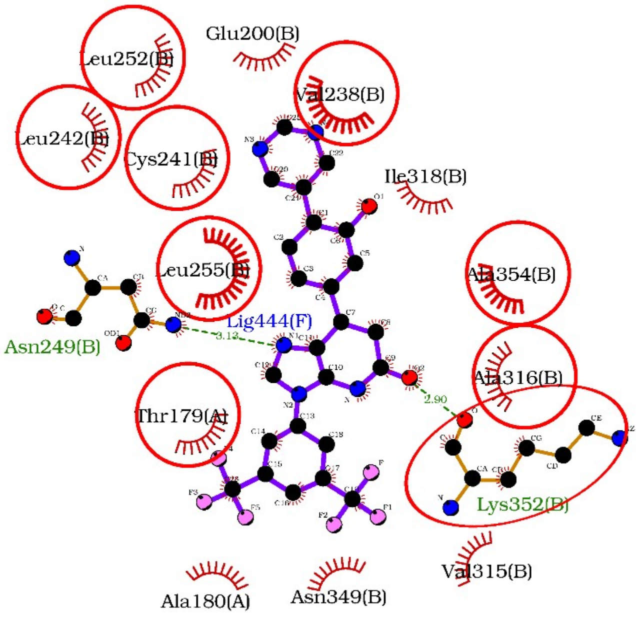

Compound 15 500 ns run 3

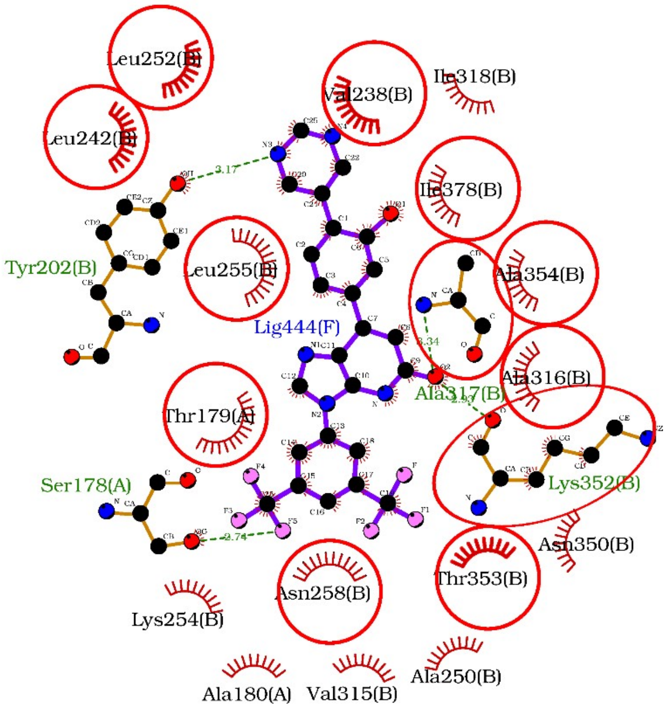

Compound 15 0 ns run 1

.

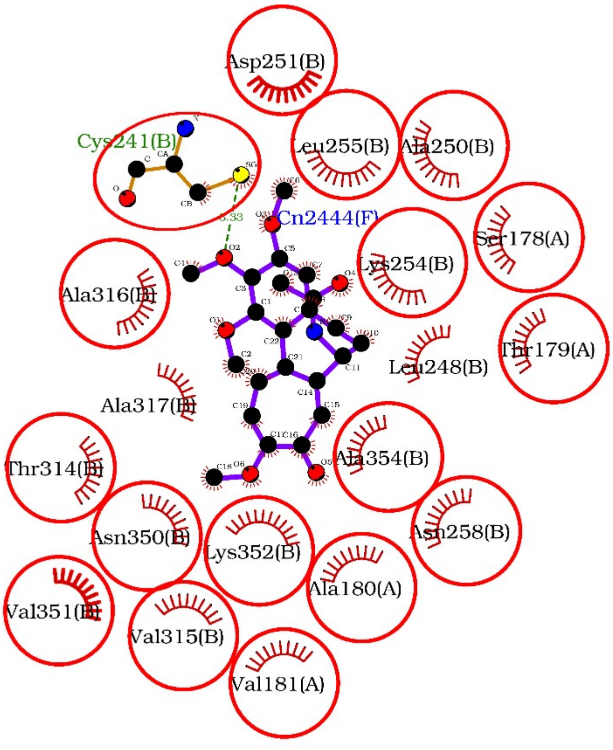

Colchicine 500 ns run 1

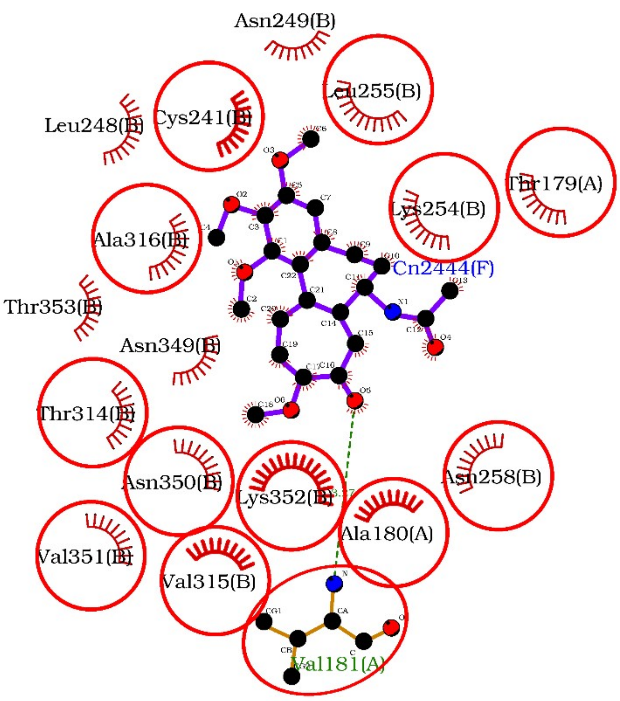

Colchicine 500 ns run 2

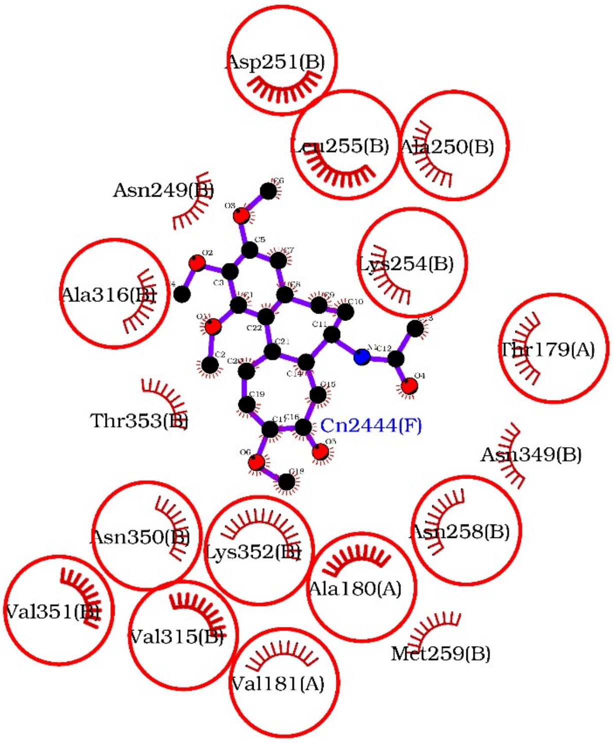

Colchicine 500 ns run 3

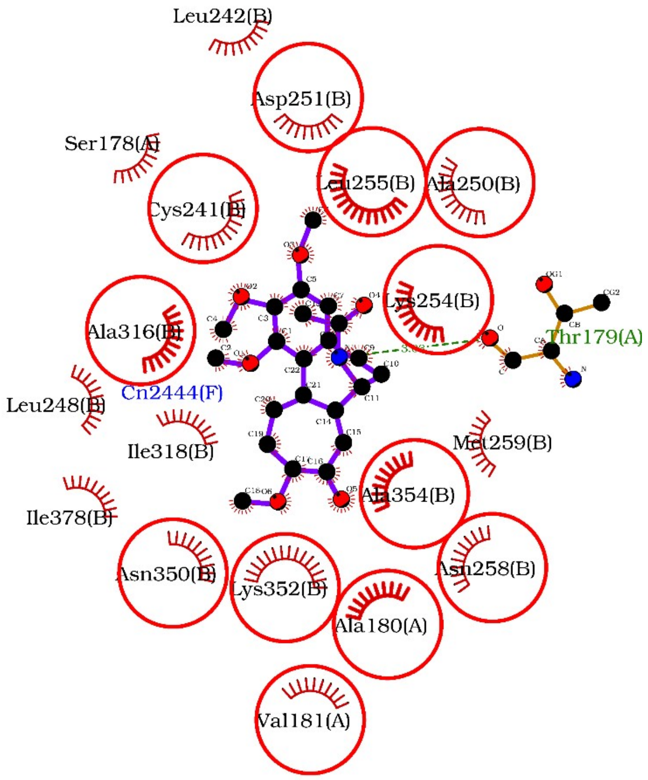

Colchicine 0 ns

Figure S5 Ligplot$^+$ diagrams of the 500 ns structures for each simulation showing ligand-residue contacts at that moment, plus their respective starting structures.

Compound 6

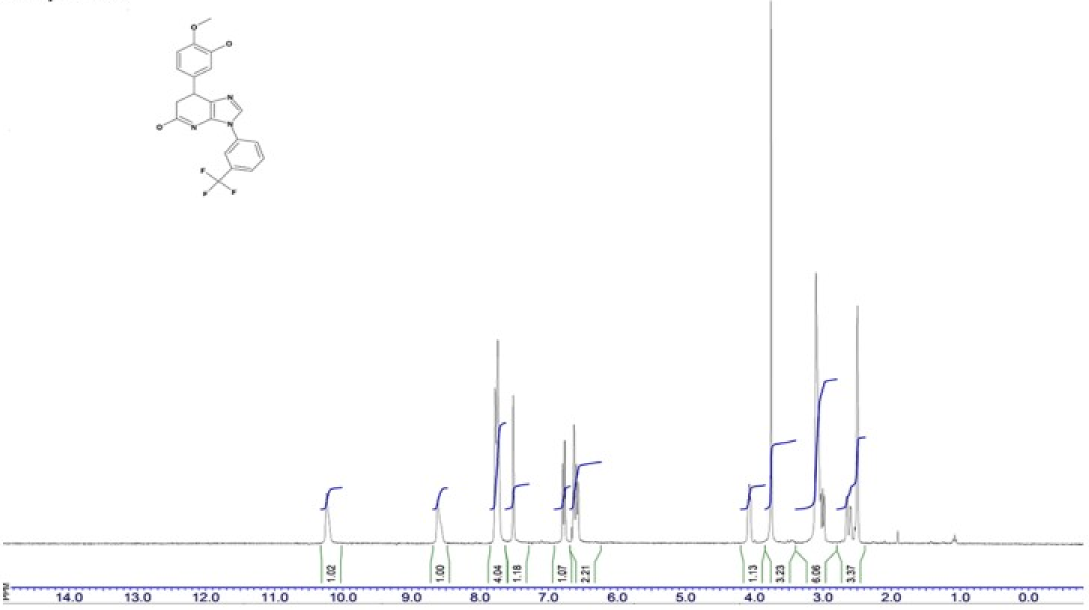

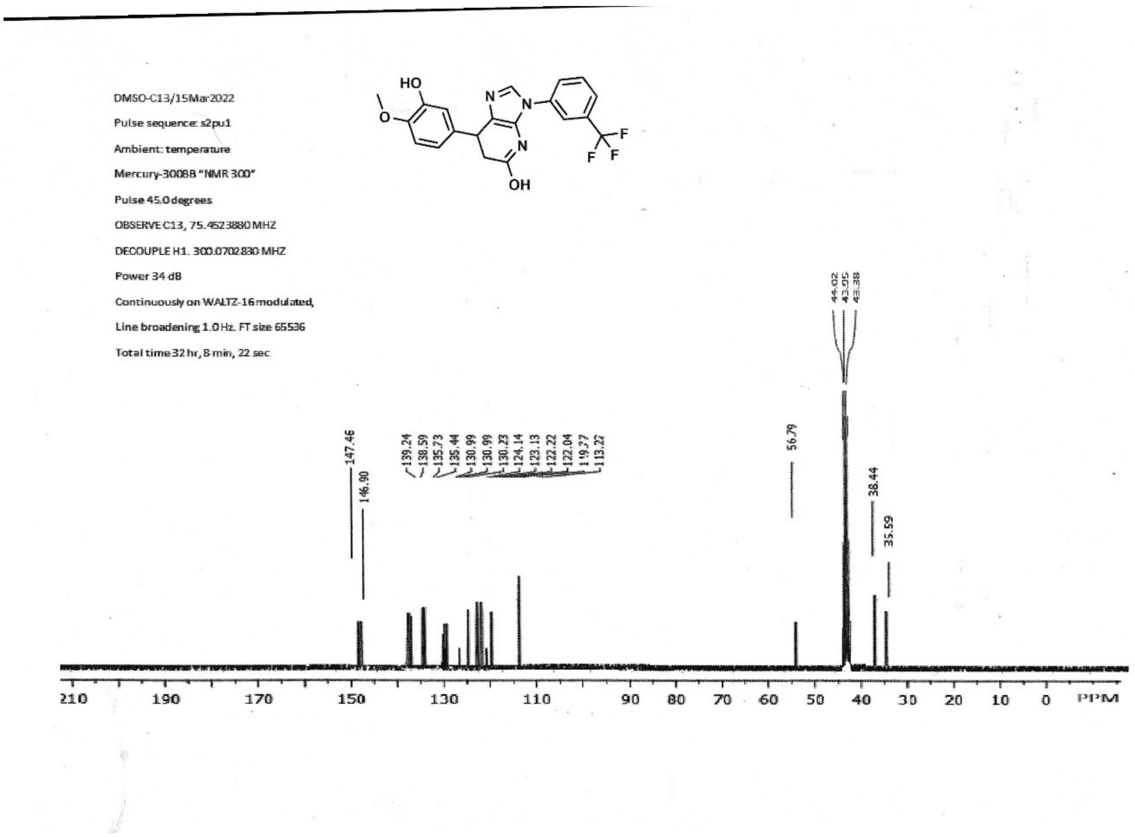

Figure S6 ^{1}H and ^{13}C NMR spectra of compound **6**.

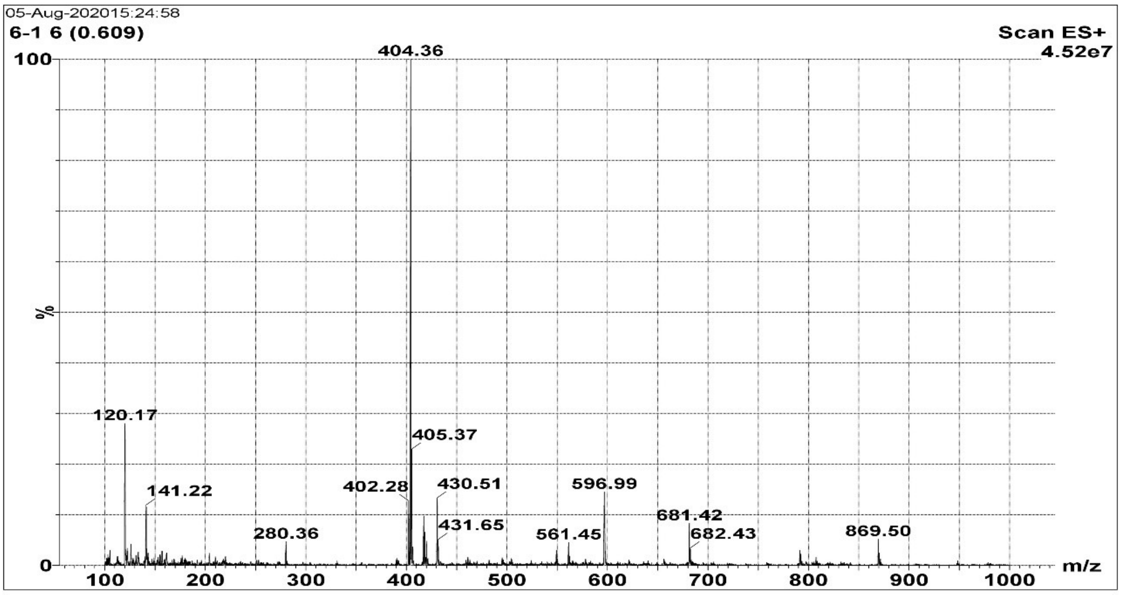

Figure S7 Mass spectrum of compound 6.

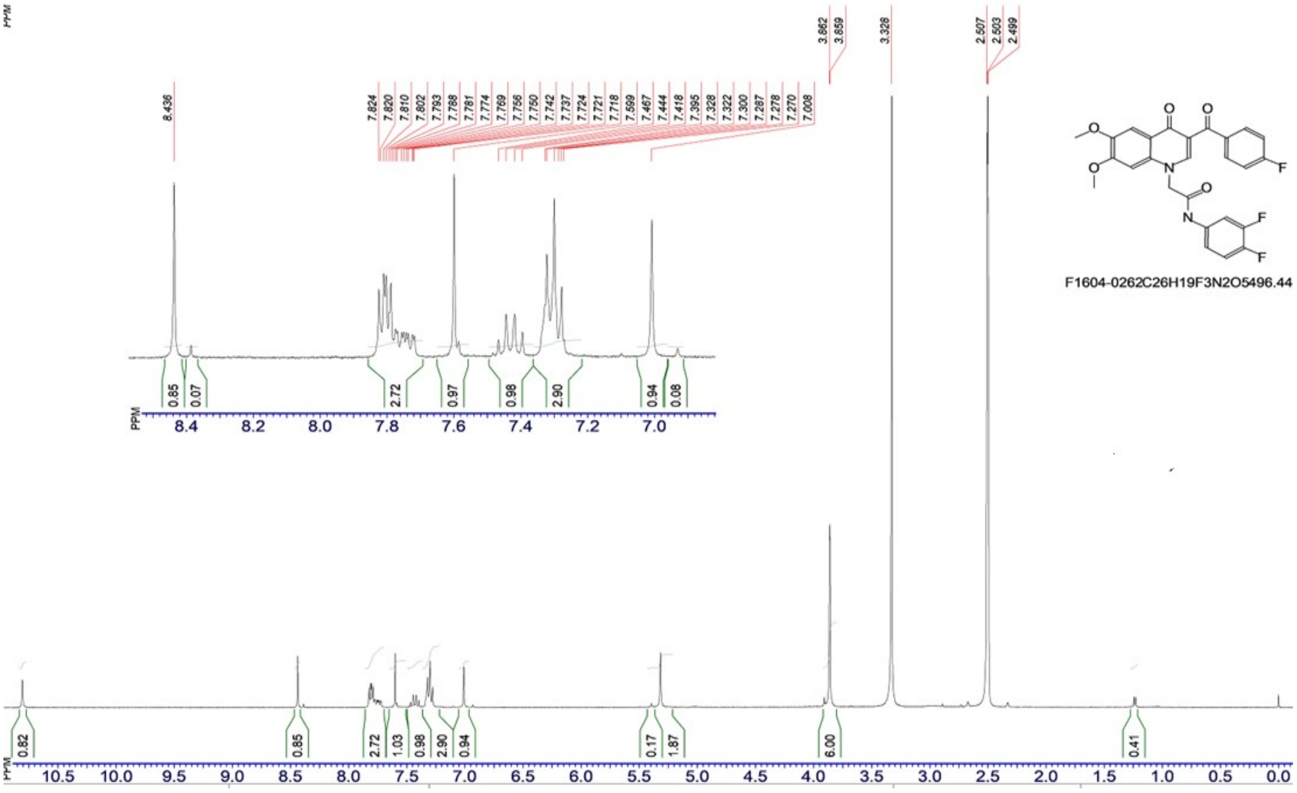

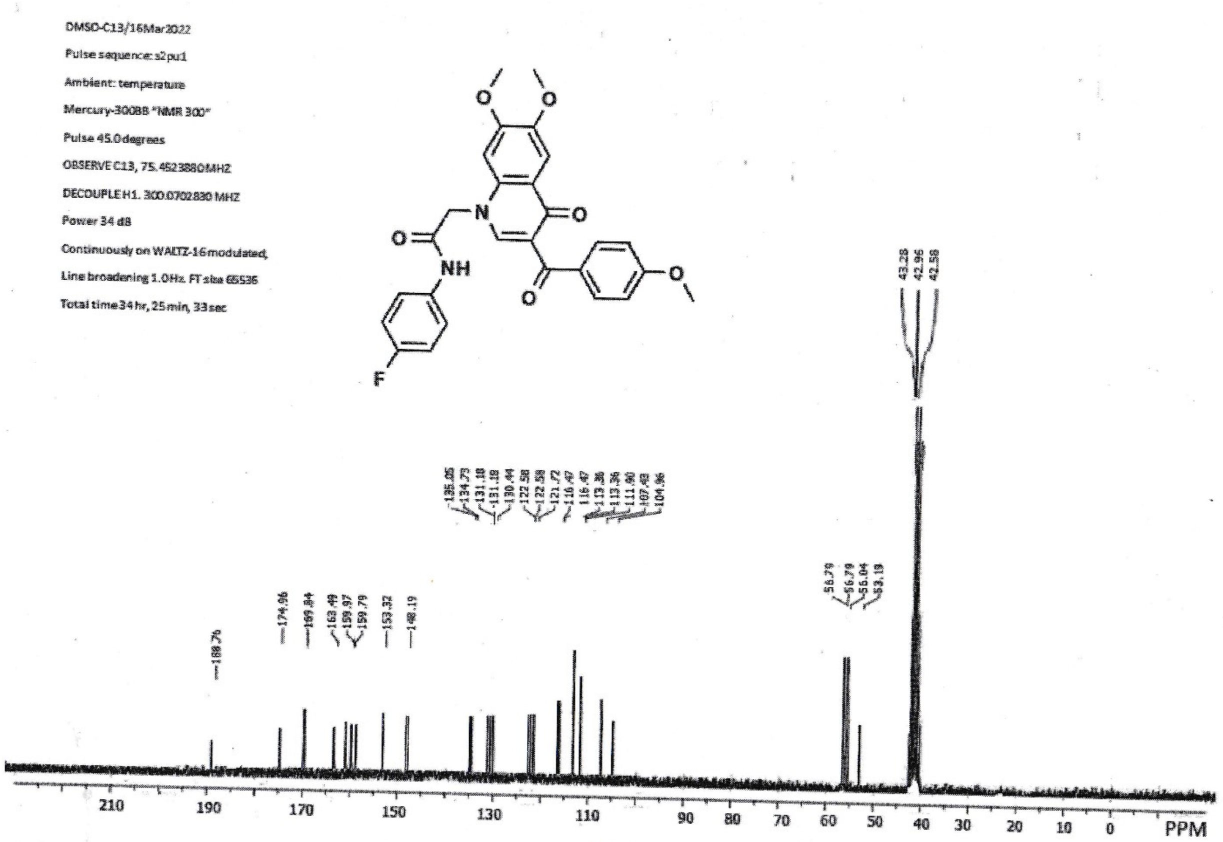

Figure S8 ^{1}H and ^{13}C NMR spectrum of compound **8**

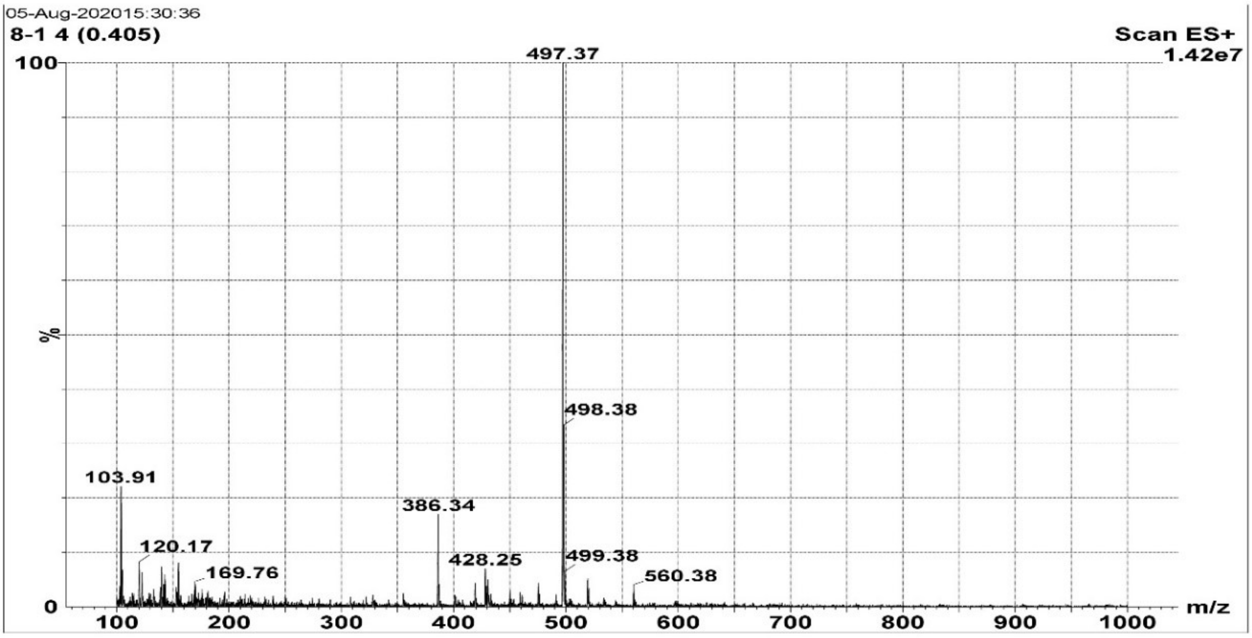

Figure S9 Mass spectrum of compound **8.**

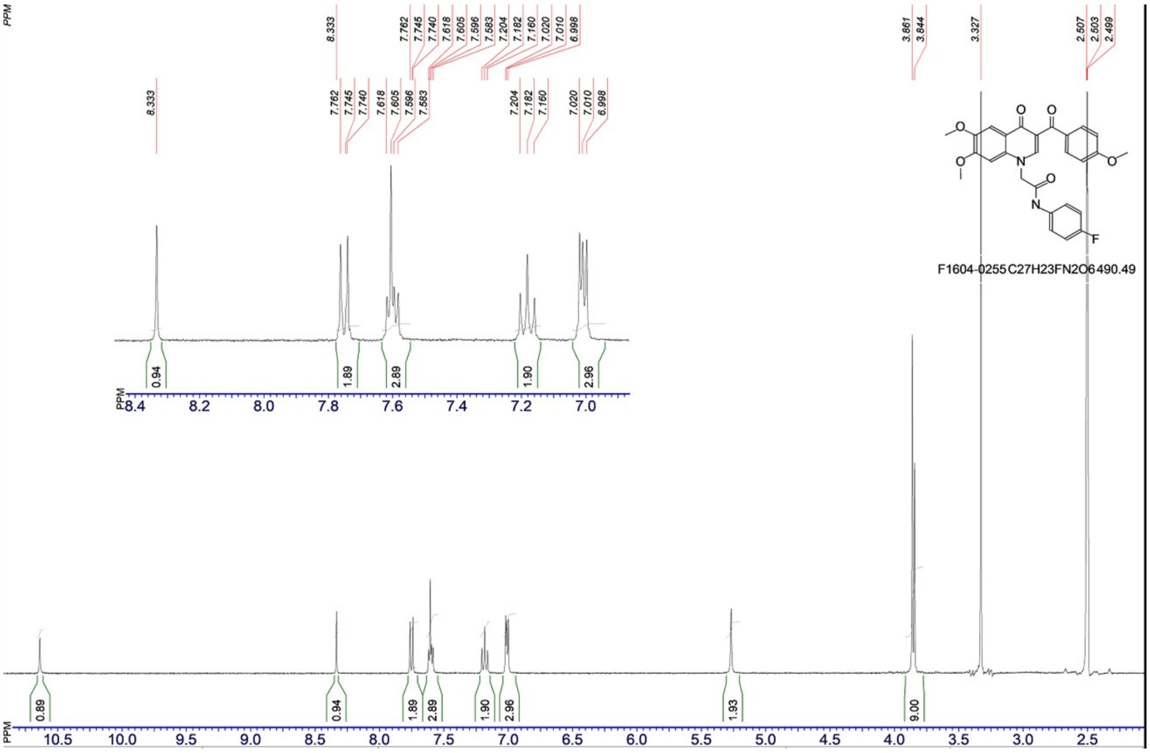

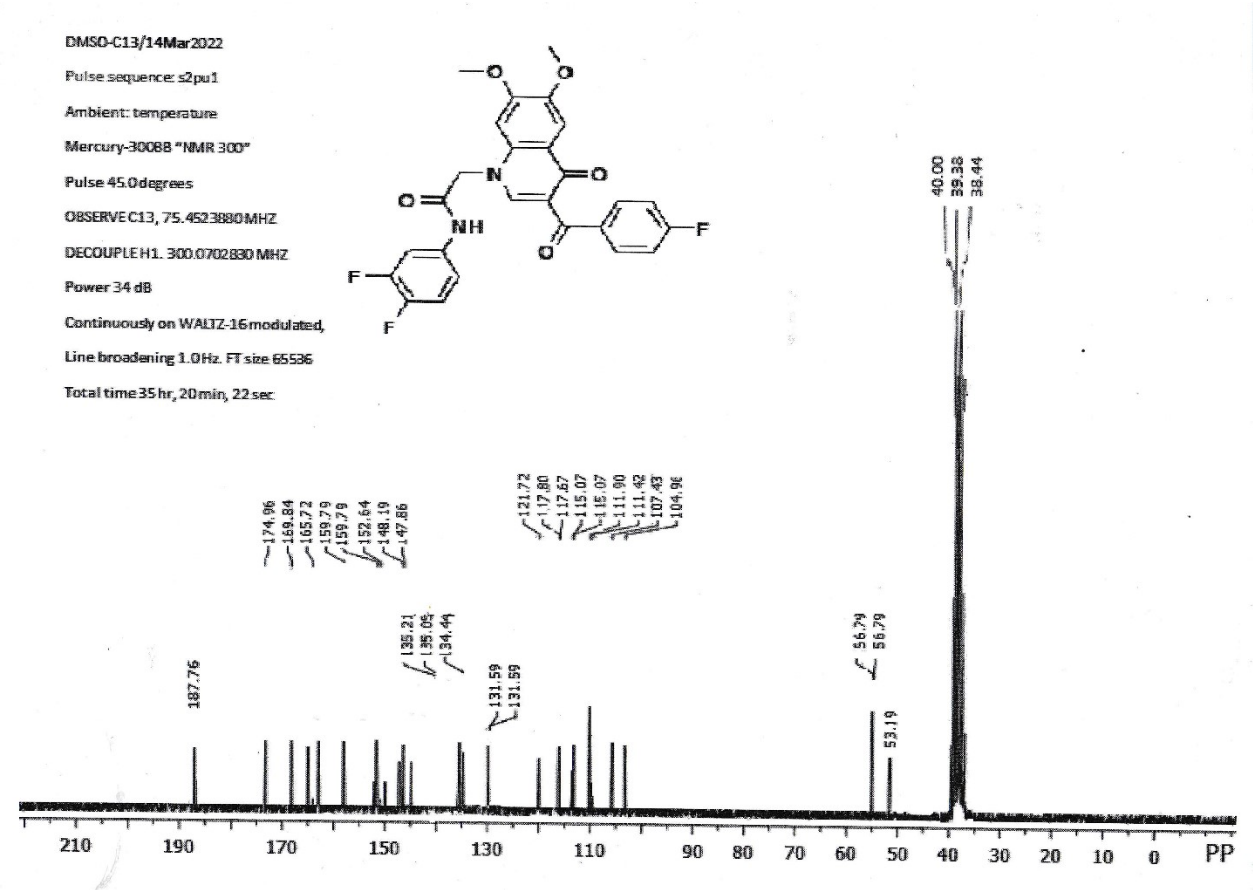

Figure S10 ^{1}H and ^{13}C NMR spectrum of compound **9**

MaxPeak: 100.00%
Ret_Time: 1.364 min

T5382903

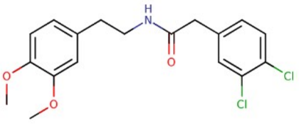

Mol Wt 368.25
Exact Mass 367.1

#	Time	Area%
1	1.364	100.00

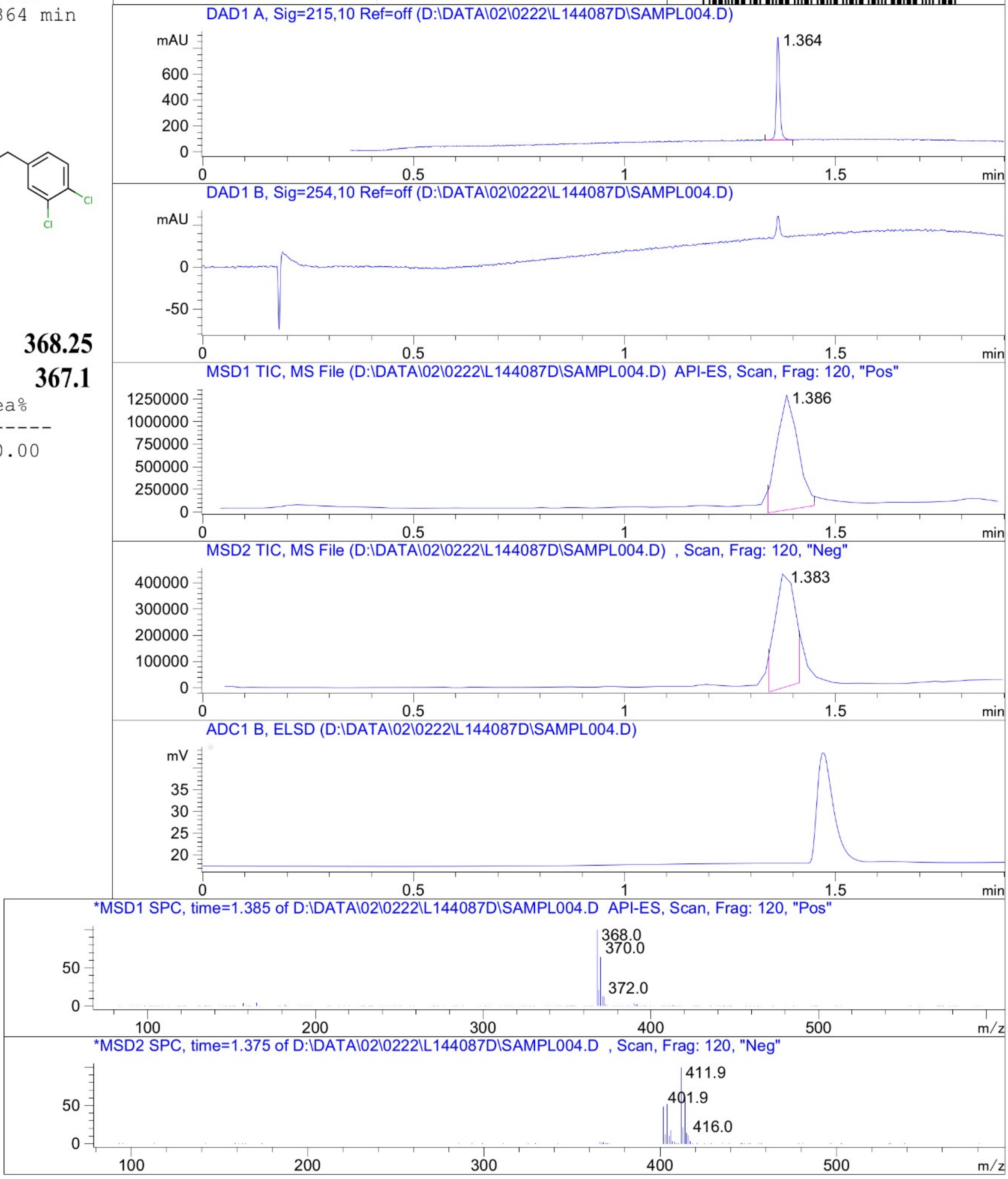

RT 1.386

RT 1.383

Inj.Date 2/22/2019 LB P2-A-04 -SL- Acq. Method C:\HPCHEM\-> ->

MaxPeak: 100.00%
Ret_Time: 1.314 min

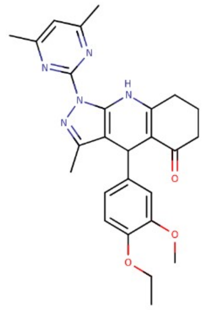

Mol Wt 459.54

Exact Mass 459.26

#	Time	Area%
1	1.314	100.00

T5105911

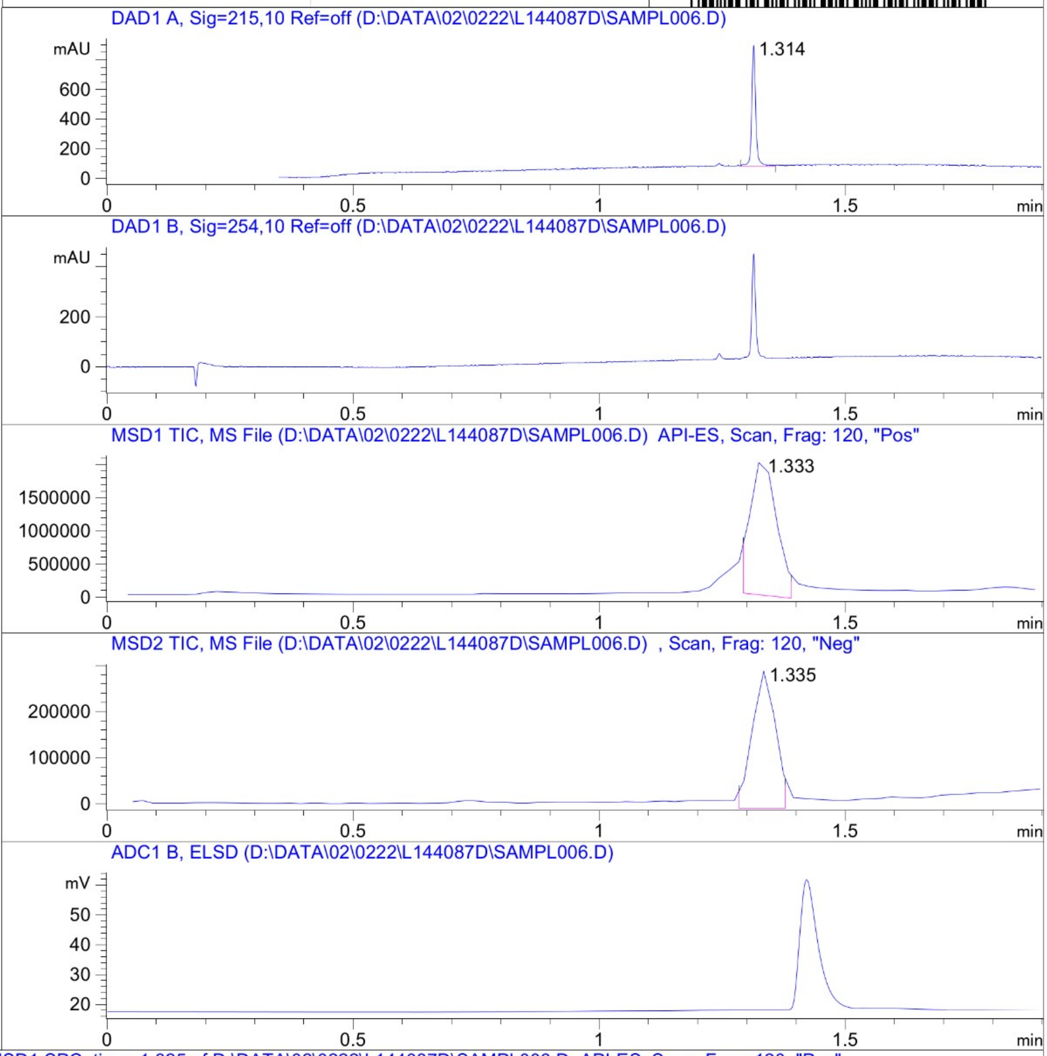

RT 1.333

RT 1.335

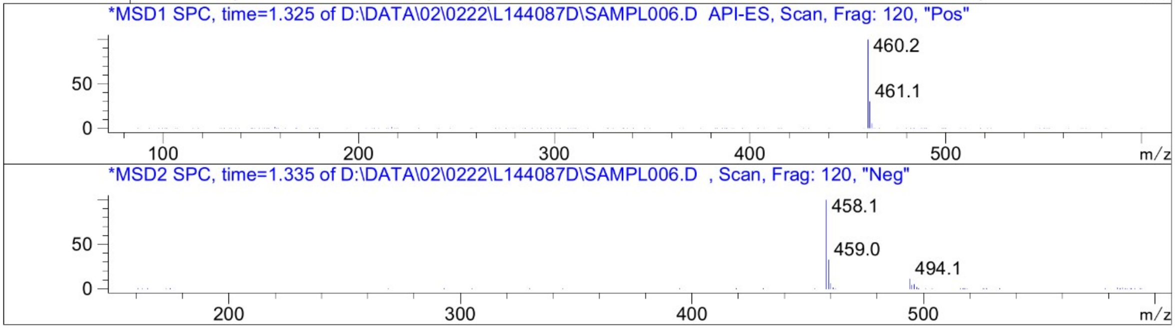

Inj.Date 2/22/2019 LB P2-A-06 -SL- Acq. Method C:\HPCHEM\-> ->

MaxPeak: 100.00%
Ret_Time: 1.183 min

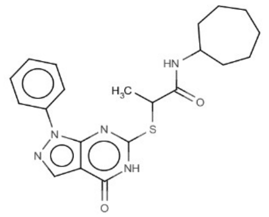

Mol Wt 411.522
Exact Mass 411.2

#	Time	Area%
1	1.183	100.00

2553300

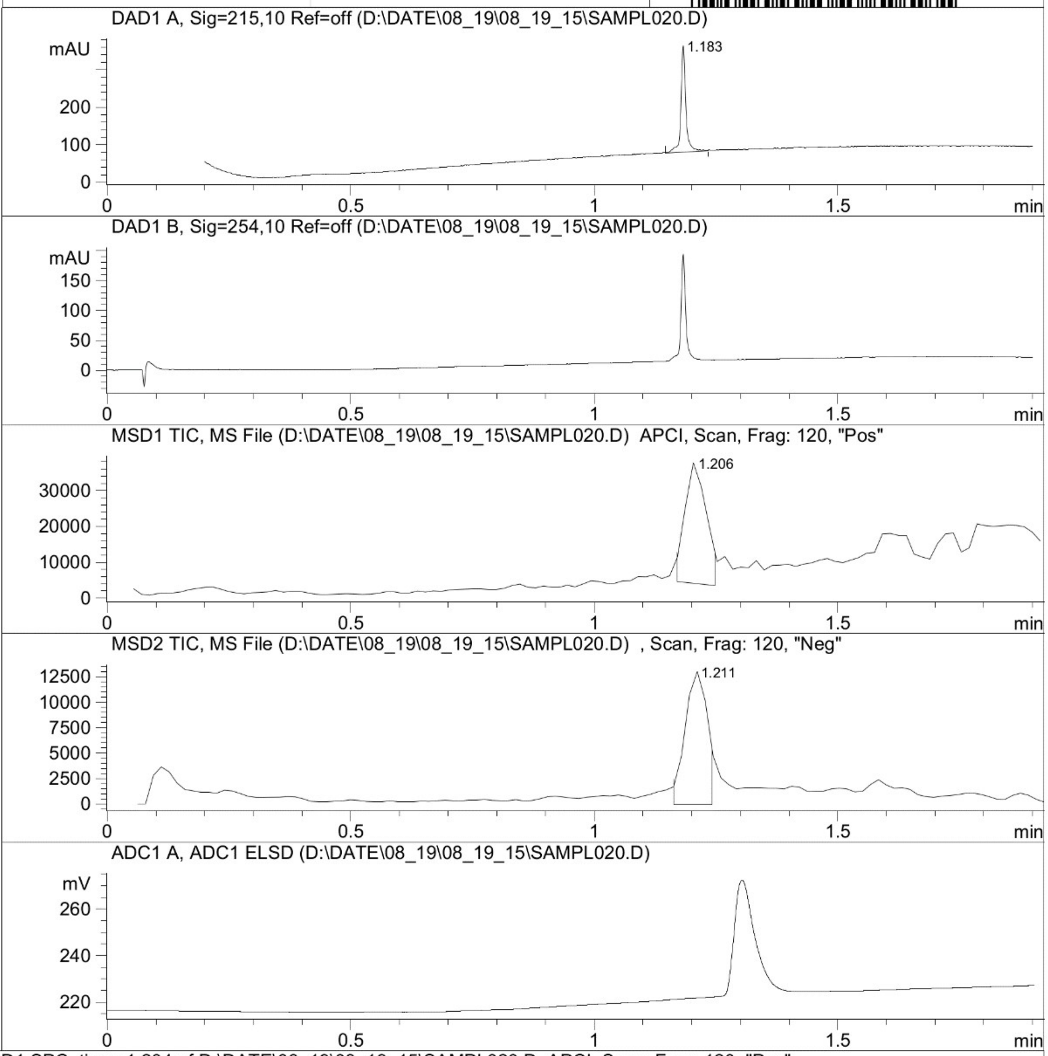

RT 1.206

RT 1.211

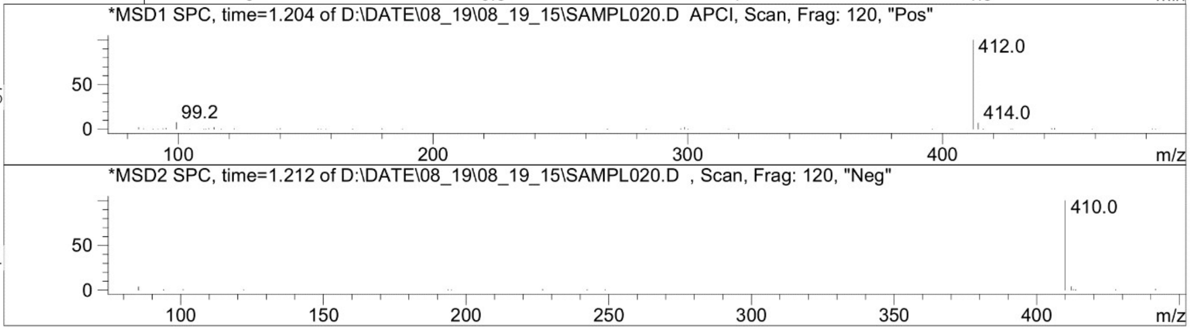

Inj.Date 8/20/2008 O P2-C-04 SL Acq. Method C:\HPCHEM\-> ->

MaxPeak: 82.46%
Ret_Time: 1.386 min

T6311120

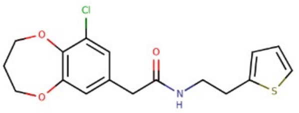

Mol Wt 351.85

Exact Mass 351.09

#	Time	Area%
1	1.128	0.41
2	1.251	0.95
3	1.366	3.41
4	1.386	82.46
5	1.420	3.06
6	1.462	1.86
7	1.525	1.11
8	1.633	5.79
9	1.707	0.55
10	1.782	0.42

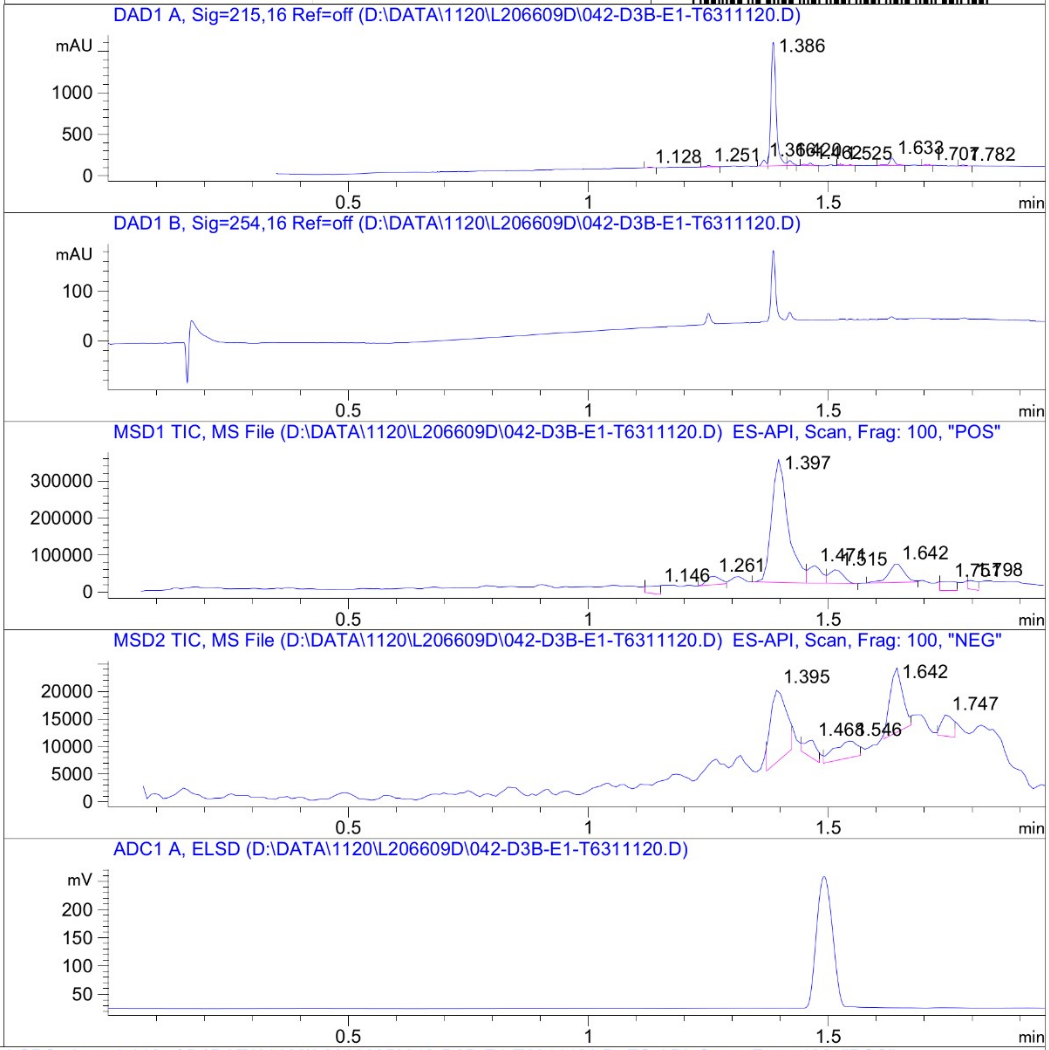

RT 1.146

RT 1.261

RT 1.397

RT 1.471

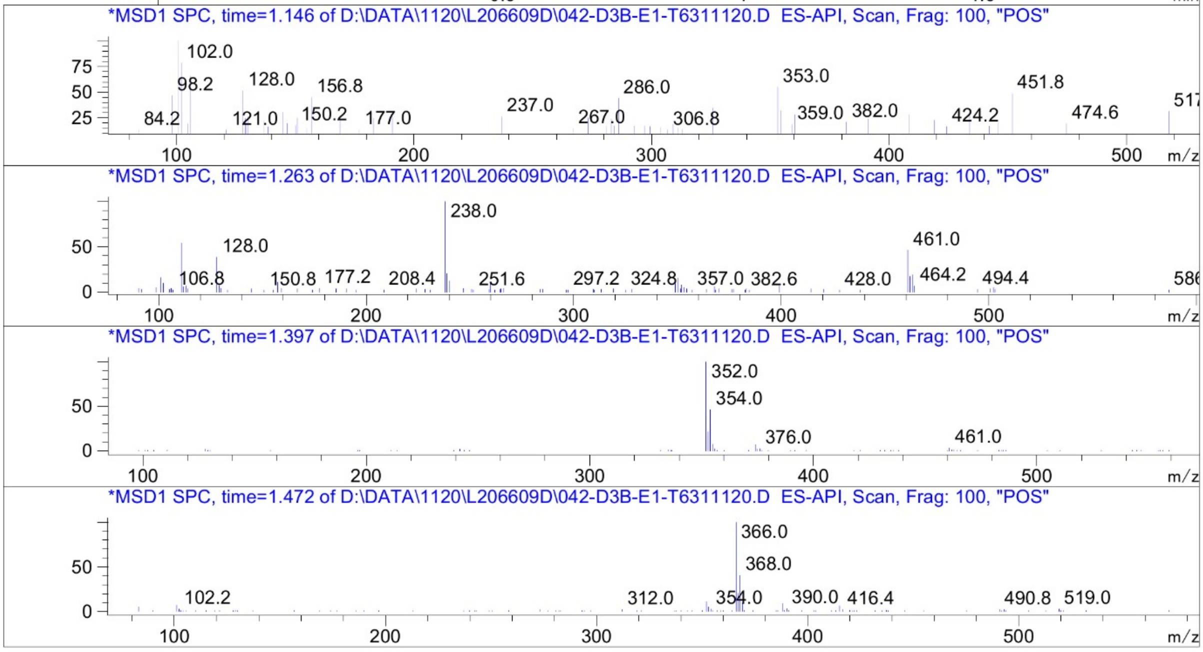

Inj.Date 11/20/2019 M <invalid> -12- Acq. Method C:\Chem32\-> ->

MaxPeak: 91.46%
Ret_Time: 1.176 min

2428744

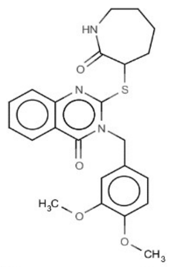

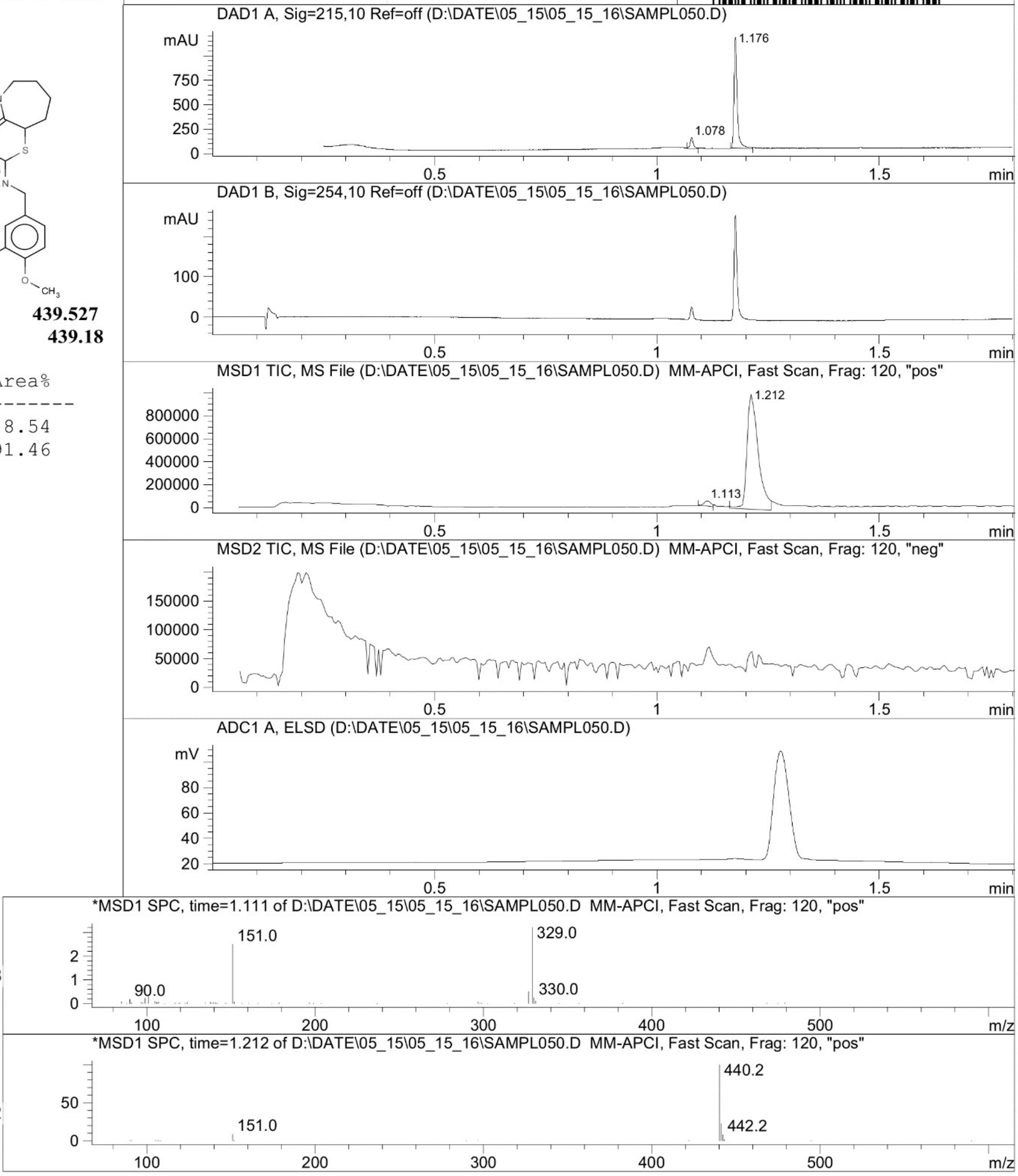

Mol Wt 439.527
Exact Mass 439.18

#	Time	Area%
1	1.078	8.54
2	1.176	91.46

RT 1.113

RT 1.212

Inj.Date 5/16/2009 O P2-F-05 RR Acq. Method C:\CHEM32\-> ->

Figure S11 Spectra and analytical date of the short-listed compounds
